# *Eurosurveillance* reviewers in 2021

**DOI:** 10.2807/1560-7917.ES.2022.27.1.2201061

**Published:** 2022-01-06

**Authors:** Eurosurveillance editorial team

**Affiliations:** 1 European Centre for Disease Prevention and Control (ECDC), Stockholm, Sweden

**Keywords:** peer-review

The editorial team is grateful to all the experts who dedicated their time to peer-review our articles over the past 12 months. We wish to thank them and all those whose names are not listed below but gave helpful advice and support. We apologise to any reviewers whose names we may have missed.

Our reviewers in 2021 were:

Cornelia Adlhoch; Marco Ajelli; Mustafa Al-Haboubi; Abdullah A Algaissi; Franz Allerberger; Erik Alm; Juan Ambrosioni; Emmi Andersson; Denise Antona; Veli-Jukka Anttila; Patricia Antunes; Ludwig Apers; Ozgur Araz; Chris Archibald; Jakob Armann; Wouter Arrazola de Oñate; Mardjan Arvand; Tommi Asikainen; Agoritsa Baka; Tamas Bakonyi; Cyril Barbezange; Lucia Barcellini; Ian Barr; Ulrike Baum; Julien Beauté; Senad Begić; Jaber Belkhiria; Leila Bell; Edward Belongia; Alexandre Belot; Cheryl Bennett; Fabian Berger; Zeno Bisoffi; Kaatje Bollaerts; Shelly Bolotin; Isabelle Bonmarin; Idesbald Boone; Timothy Booth; Robert Booy; Maria José Borrego; Michael Brandl; Arne Brantsæter; Jan Brauner; David Brett-Major; Anne Brisabois; Tom Britton; Norbert H. Brockmeyer; Kim Brolin; Jesca Brouwer; Alison Brown; Colin Brown; Udo Buchholz; Silke Buda; Niccolò Buetti; Karina Butler; Zahid Butt; Merle Margarete Böhmer; Saverio Caini; Sébastien Calvignac-Spencer; Yehuda Carmeli; Eugenia Carrillo; Anne Carroll; Carlos Carvalho; Kelsie Cassell; Jesus Castilla Catalan; Simon Cauchemez; Dominique Caugant; Marco Cavaleri; Nathalie Charlotte; Remi Charrel; Tianmu Chen; Edward Choi; Eun Hwa Choi; Gerardo Chowell; Konstantin Chumakov; Annarita Ciccaglione; Heike Claus; Noelle Cocoros; Michelle Cole; Vittoria Colizza; Alex Cook; Geoffrey Coombs; Victor Corman; Laura Cornelissen; Ana Maria Azevedo Correia; Suzanne Cotter; Benjamin Cowling; Keith Crandall; Natasha Crowcroft; Fortunato D'Ancona; Gavin Dabrera; Andrei Dadu; Stephanie Dancer; Nicholas Davies; Brechje de Gier; Gaston De Serres; Gerard de Vries; Walter Demczuk; Aleksander Deptula; Phillippe Dewals; Vijaykrishna Dhanasekaran; Laura Di Domenico; Yaakov Dickstein; Joanne Dillon; Jac Dinnes; Lisa Domegan; Gé Donker; Yvonne Doyle; Aparna Dressler; Jan Drexler; Timothee Dub; Sebastian Duchene; Erwin Duizer; Dominic Dwyer; Robert Dyrdak; Michael Edelstein; Dirk Eggink; Hanne-Dorthe Emborg; Theresa Enkirch; Mirko Faber; Christopher Fairley; Luzhao Feng; Helen Fifer; Julie Figoni; Bastian Fischer; Brendan Flannery; Jannik Fonager; Arnaud Fontanet; Christina Frank; Ana Isabel Freitas; Luca Freschi; Ingrid Friesema; Shouichi Fujimoto; Emmanouil Galanakis; Irene Galani; Amiran Gamkrelidze; Karthik Gangavarapu; Patricia Garcia de Olalla; Patricia Garvey; Andrea Gervelmaeyer; Pere Godoy; Richard V. Goering; Edward Goldstein; Rok Grah; Devon Greyson; Roland Grunow; Thorolfur Gudnason; Claire Guinat; Rachel Gur-Arie; Jean-Paul Guthmann; Eric Haas; Walter Haas; Susan Hahne; Wanda Han; Pia Hardelid; Thomas Harder; David M. Hartley; Heli Harvala; Naoki Hasegawa; Joana Haussig; Qiushui He; Kristin Hegstad; Janneke Heijne; Wolfram Henn; Jean-Michel Heraud; Victoria Hernando; Thomas Hofmann; Yingfen Hsia; Jim F Huggett; Gwenda Hughes; Hilary Humphreys; Benedikt Huttner; Stefano Iacus; Derval Igoe; Michael G Ison; Michael Jackson; Stine Jakobsen; Sanjay Jayasinghe; David Jenkins; Cecilia Jernberg; Silvia Jimenez-Jorge; Kari Johansen; Tone Johansen; Jerker Jonsson; Frédéric Jourdain; Irena Jukić; Jaehun Jung; Annemarjut Jääskeläinen; Sarah Kabbani; Oliver Kacelnik; Kamran Kadkhoda; Bernard Kaic; Taishi Kayano; Winfried Kern; Ani Kevorkyan; Marian Killip; John Kinsman; Amir Kirolos; Esther Kissling; Jonas Klingström; Marian Knight; Mirjam Knol; Anke Kohlenberg; Philipp Kohler; Axel Kola; Rolf Kramer; Annette Kraus; Gerard Krause; Manuel Krone; Andreas Kronenberg; Satu Kurkela; Jeff Kwong; Theresa Lamagni; Amparo Larrauri; Kevin B Laupland; Quentin Leclerc; Shui-Shan Lee; Clara Lehmann; Kathy Leung; Lanjuan Li; Pieter Libin; Andreas Lieberoth; Bruno Lina; Florence Lot; Yaniv Lustig; Michael F. Lynch; Frederik Lyngse; Theodore Lytras; Outi Lyytikäinen; Yiqun Ma; Gannon C.K. Mak; Michal Mandelboim; Davide Manissero; Elisa Martín-Merino; Josefa Masa-Calles; Alberto Mateo Urdiales; Alberto Matteelli; Kathleen Mazor; Andrew McAuley; Andrea McCollum; Jim McMenamin; Eleanor Mcnamara; Michael McNeil; Gertjan Medema; Paula Meireles; Angeliki Melidou; Stefano Merler; Devon S Metcalf; Benjamin Meyer; Susana Monge; Dominique Monnet; Orna Mor; Roger Morbey; Alain Moren; Pedro l. Moro; Joël Mossong; Judith Mueller; Kristine Mørch; Marcel Müller; Sophie Nash; Eleni Nastouli; Pontus Naucler; Ndeindo Ndeikoundam Ngangro; Elsa Negro Calduch; Richard Neher; Nathalie Nicolay; Hiroshi Nishiura; Andreas Nitsche; Harold Noel; Robert Noland; Daan Notermans; Micha Nübling; Joan O'Donnell; Charlotte O'Halloran; Steve Oberste; Kazunori Oishi; Jason Ong; David Pace; W Paget; Nitika Pai; Scott Pallett; Takis Panagiotopoulos; Elisabetta Pandolfi; Junxiong Vincent Pang; Annalisa Pantosti; Dimitrios Paraskevis; Milosz Parczewski; Maria Pardos de la Gandara; Minal Patel; Samir Patel; Maria Paulke-Korinek; Pasi Penttinen; André Peralta-Santos; Javier Perez-Saez; John Pettersson; Naomi Petty-Saphon; Tung Phan; leanne pillay; Adriana Pistol; Denis Piérard; Diamantis Plachouras; Laurent Poirel; Jose A Ruiz Postigo; Isabelle Poujol; Wolfgang Preiser; ‪Giovambattista Presti; Katarina Prosenc; Joan Puig-Barbera; Annalisa Quattrocchi; Sophie Quoilin; Public Rajatanavin; Venerando Rapisarda; Giovanni Ravasi; Carrie Reed; Ralf Reintjes; Ute Rexroth; Samuel Rhedin; Maria Luisa Ricci; Maximilian Riess; Michael Rigby; Thomas V. Riley; Julien Riou; Lorenz Risch; Annapaola Rizzoli; Emmanuel Robesyn; Marc Rondy; Werner Ruppitsch; Timothy Russell; Rachel Sacks-Davis; Katilyn Sadtler; Donald Salami; Paula Salmeron; Jet Sanders; Roy Sanderson; Frank Sandmann; Lawrence Sawchuk; Gianpaolo Scalia-Tomba; Samuel Scarpino; Astrid Blicher Schelde; Emily Scher; David Schwartz; Tatjana Schwarz; Matthew Scotch; Melissa Sharp; Lester Shulman; Reina Sikkema; Ryan Simpson; Vitali Sintchenko; Mary P. E. Slack; Morgane Solis; Gianfranco Spiteri; Megan Steain; Chen Stein-Zamir; Silvia Stringhini; Heino Stöver; Lorenzo Subissi; Carl Suetens; Jonathan Suk; Barbara Suligoi; Patrick Sullivan; Sheena Sullivan; Pål Suren; Pham Thi Mui; Daniel Thirion; Bruce Thorley; Karen Tiley; Jean-François Timsit; Kelvin To; Maria Elena Tosti; Alberto Tozzi; Svetla Tsolova; Masaru Usui; Sophie Valkenburg; Maarten van Cleeff; Kees. C. van den Wijngaard; Marianne van der Sande; Esther Van Kleef; Thomas Vanwolleghem; Olli Vapalahti; Maria Louise Veimer Jensen; Lasse Vestergaard; Pauline Vetter; Jorge Vicente-Guijarro; Viktor von Wyl; Rengina Vorou; Jacco Wallinga; Michelle Waltenburg; Qiang Wang; Daniel Weinberger; Christian Wejse; Matthijs Welkers; Chard Richard Wells; Heather Whitaker; Andreas Widmer; Ursula Wiedermann; Annelies Wilder-Smith; Eloise Williams; Thomas Williams; Emma Wiltshire; Brita Askeland Winje; Carl-Heinz Wirsing von König; Shell Wong; Takuya Yamagishi; Hsin-Chou Yang; Shangxin Yang; Qing Ye; Dominik Zenner; Walter Zingg; Phillip Zucs.

